# The Appalachia Mind Health Initiative (AMHI): a pragmatic randomized clinical trial of adjunctive internet-based cognitive behavior therapy for treating major depressive disorder among primary care patients

**DOI:** 10.1186/s13063-022-06438-y

**Published:** 2022-06-20

**Authors:** Robert M. Bossarte, Ronald C. Kessler, Andrew A. Nierenberg, Ambarish Chattopadhyay, Pim Cuijpers, Angel Enrique, Phyllis M. Foxworth, Sarah M. Gildea, Bea Herbeck Belnap, Marc W. Haut, Kari B. Law, William D. Lewis, Howard Liu, Alexander R. Luedtke, Wilfred R. Pigeon, Larry A. Rhodes, Derek Richards, Bruce L. Rollman, Nancy A. Sampson, Cara M. Stokes, John Torous, Tyler D. Webb, Jose R. Zubizarreta

**Affiliations:** 1 Present Address: Department of Psychiatry and Behavioral Neuroscience, University of South Florida, 3515 E. Fletcher Ave, FL 33613 Tampa, USA; 2 Department of Healthcare Policy, Harvard Medical School, Boston, MA USA; 3 The Dauten Family Center for Bipolar Treatment Innovation, Massachusetts General Hospital and Harvard Medical School, Boston, MA USA; 4 Department of Statistics, Harvard University, Cambridge, USA; 5 Department of Clinical, Neuro and Developmental Psychology, Amsterdam Public Health Research Institute, Vrije Universiteit Amsterdam, Van der Boechorststraat 7-9, Amsterdam, 1081 BT The Netherlands; 6 E-mental Health Research Group, School of Psychology, University of Dublin, Trinity College Dublin and Clinical Research & Innovation, SilverCloud Health, Dublin, Ireland; 7 Depression and Bipolar Support Alliance, Chicago, IL USA; 8 Department of Health Care Policy, Harvard Medical School, Boston, MA USA; 9 Center for Behavioral Health, Media, and Technology, University of Pittsburgh School of Medicine, Pittsburgh, PA USA; 10 Department of Behavioral Medicine and Psychiatry, West Virginia University School of Medicine, Morgantown, WV USA; 11 Department of Neurology, West Virginia University School of Medicine, Morgantown, WV USA; 12 Department of Radiology, West Virginia University School of Medicine, Morgantown, WV USA; 13 Department of Family Medicine, West Virginia University School of Medicine and West Virginia University Clinical and Translational Science Institute, Morgantown, WV USA; 14 Center of Excellence for Suicide Prevention, Canandaigua VA Medical Center, Canandaigua, NY USA; 15 Department of Statistics, University of Washington and Vaccine and Infectious Disease Division, Fred Hutchinson Cancer Research Center, Seattle, WA USA; 16 Department of Psychiatry, University of Rochester Medical Center, Rochester, NY 14642 USA; 17 Department of Pediatrics, West Virginia University School of Medicine and West Virginia University Institute for Community and Rural Health, Morgantown, WV USA; 18 West Virginia University Injury Control Research Center, Morgantown, WV USA; 19 Department of Psychiatry, Beth Israel Deaconess Medical Center, Harvard Medical School, Boston, MA USA; 20 Department of Statistics, Harvard University, Cambridge, MA USA; 21 Department of Biostatistics, Harvard University, Cambridge, MA USA

**Keywords:** Appalachian Mind Health Initiative (AMHI), Major depressive disorder, i-CBT, Remission from depression, Antidepressant medication, Heterogeneity of treatment effects

## Abstract

**Background:**

Major depressive disorder (MDD) is a leading cause of disease morbidity. Combined treatment with antidepressant medication (ADM) plus psychotherapy yields a much higher MDD remission rate than ADM only. But 77% of US MDD patients are nonetheless treated with ADM only despite strong patient preferences for psychotherapy. This mismatch is due at least in part to a combination of cost considerations and limited availability of psychotherapists, although stigma and reluctance of PCPs to refer patients for psychotherapy are also involved. Internet-based cognitive behaviorial therapy (i-CBT) addresses all of these problems.

**Methods:**

Enrolled patients (*n* = 3360) will be those who are beginning ADM-only treatment of MDD in primary care facilities throughout West Virginia, one of the poorest and most rural states in the country. Participating treatment providers and study staff at West Virginia University School of Medicine (WVU) will recruit patients and, after obtaining informed consent, administer a baseline self-report questionnaire (SRQ) and then randomize patients to 1 of 3 treatment arms with equal allocation: ADM only, ADM + self-guided i-CBT, and ADM + guided i-CBT. Follow-up SRQs will be administered 2, 4, 8, 13, 16, 26, 39, and 52 weeks after randomization. The trial has two primary objectives: to evaluate aggregate comparative treatment effects across the 3 arms and to estimate heterogeneity of treatment effects (HTE). The primary outcome will be episode remission based on a modified version of the patient-centered Remission from Depression Questionnaire (RDQ). The sample was powered to detect predictors of HTE that would increase the proportional remission rate by 20% by optimally assigning individuals as opposed to randomly assigning them into three treatment groups of equal size.

Aggregate comparative treatment effects will be estimated using intent-to-treat analysis methods. Cumulative inverse probability weights will be used to deal with loss to follow-up. A wide range of self-report predictors of MDD heterogeneity of treatment effects based on previous studies will be included in the baseline SRQ. A state-of-the-art ensemble machine learning method will be used to estimate HTE.

**Discussion:**

The study is innovative in using a rich baseline assessment and in having a sample large enough to carry out a well-powered analysis of heterogeneity of treatment effects. We anticipate finding that self-guided and guided i-CBT will both improve outcomes compared to ADM only. We also anticipate finding that the comparative advantages of adding i-CBT to ADM will vary significantly across patients. We hope to develop a stable individualized treatment rule that will allow patients and treatment providers to improve aggregate treatment outcomes by deciding collaboratively when ADM treatment should be augmented with i-CBT.

**Trial registration:**

ClinicalTrials.gov NCT04120285. Registered on October 19, 2019.

## Administrative information

Note: the numbers in curly brackets in this protocol refer to SPIRIT checklist item numbers. The order of the items has been modified to group similar items (see http://www.equator-network.org/reporting-guidelines/spirit-2013-statement-defining-standard-protocol-items-for-clinical-trials/).

**Table:** 

**Title {1}**	**The Appalachian Mind Health Initiative (AMHI): A pragmatic clinical trial of adjunctive internet-based Cognitive Behavior Therapy for treating major depressive disorder among primary care patients**
**Trial registration {2a and 2b}.**	ClinicalTrials.gov Identifier: NCT04120285
**Protocol version {3}**	Initial version date: 10/15/20
**Funding {4}**	This trial is funded by the Patient Centered Outcomes Research Institute (PCORI).
**Author details {5a}**	Robert M. Bossarte, PhD; West Virginia University Injury Control Research Center and Department of Behavioral Medicine and Psychiatry, West Virginia University School of Medicine, Morgantown, WV Ronald C. Kessler, PhD; Department of Healthcare Policy, Harvard Medical School, Boston, MA Andrew A. Nierenberg, MD; The Dauten Family Center for Bipolar Treatment Innovation, Massachusetts General Hospital and Harvard Medical School, Boston, MA Pim Cuijpers, PhD; Department of Clinical, Neuro and Developmental Psychology, Amsterdam Public Health research institute, Vrije Universiteit Amsterdam, Van der Boechorststraat 7-9, 1081 BT, Amsterdam, The Netherlands Angel Enrique, PhD; E-mental Health Research Group, School of Psychology, University of Dublin, Trinity College Dublin and Clinical Research & Innovation, SilverCloud Health, Dublin, Ireland Phyllis M. Foxworth, BS; Depression and Bipolar Support Alliance, Chicago, IL Sarah M. Gildea, BS; Department of Health Care Policy, Harvard Medical School, Boston, Massachusetts Bea Herbeck Belnap, PhD; Center for Behavioral Health, Media, and Technology, University of Pittsburgh School of Medicine Marc W. Haut, PhD; Department of Behavioral Medicine and Psychiatry, West Virginia University School of Medicine, Department of Neurology, West Virginia University School of Medicine and Department of Radiology, West Virginia University School of Medicine, Morgantown, WV Kari B. Law, MD; Department of Behavioral Medicine and Psychiatry, West Virginia University School of Medicine, Morgantown, WV William D. Lewis, MD; Department of Family Medicine, West Virginia University School of Medicine and West Virginia University Clinical and Translational Science Institute, Morgantown, WV Howard Liu, SD; Department of Health Care Policy, Harvard Medical School, Boston, MA and Center of Excellence for Suicide Prevention, Canandaigua VA Medical Center, Canandaigua, NY Alexander R. Luedtke, PhD; Department of Statistics, University of Washington and Vaccine and Infectious Disease Division, Fred Hutchinson Cancer Research Center, Seattle, WA Wilfred R. Pigeon, PhD; Center of Excellence for Suicide Prevention, Canandaigua VA Medical Center, Canandaigua, NY and Department of Psychiatry, University of Rochester Medical Center, Rochester, NY 14642 USA Larry A. Rhodes, MD; Department of Pediatrics, West Virginia University School of Medicine and West Virginia University Institute for Community and Rural Health, Morgantown, WV Derek Richards, PhD; E-mental Health Research Group, School of Psychology, University of Dublin, Trinity College Dublin and Clinical Research & Innovation, SilverCloud Health, Dublin, Ireland Bruce L. Rollman, MD, MPH; Center for Behavioral Health, Media and Technology, University of Pittsburgh, Pittsburgh, PA Nancy A. Sampson, BA; Department of Health Care Policy, Harvard Medical School, Boston, Massachusetts Cara M. Stokes, PhD; West Virginia University Injury Control Research Center and Department of Behavioral Medicine and Psychiatry, West Virginia University School of Medicine, Morgantown, WV John Torous, MD; Department of Psychiatry, Beth Israel Deaconess Medical Center, Harvard Medical School, Boston, Massachusetts Tyler D. Webb, MSW; West Virginia University Injury Control Research Center, Morgantown, WV Jose R. Zubizarreta, PhD; Department of Health Care Policy, Harvard Medical School, Boston, MA, Department of Statistics, Harvard University and Department of Biostatistics, Harvard University, Cambridge, MA,
**Name and contact information for the trial sponsor {5b}**	Patient Centered Outcomes Research Institute (PCORI) 1828 L Street NW Suite 900 Washington, DC 20036 202-683-6690
**Role of sponsor {5c}**	PCORI funds this trial. The funder is not involved in study design, study execution, writing of reports or the decision to submit reports for publication.

## Introduction

### Background and rationale {6a}

Major depressive disorder (MDD) is one of the most burdensome of all disorders [1]. Indeed, the Global Burden of Disease study ranks MDD as the 2nd top cause of disease morbidity in the USA [2] due to the combination of its high prevalence and high impairment. MDD is associated with high work disability, absenteeism, and lost work productivity [3] and is also a powerful risk factor for suicide [4]. Based on these results, the annual economic burden of MDD in the USA is estimated to be $210 billion [5], but this estimate omits indirect costs such as associations of MDD with increased risk of subsequent onset [6] of chronic physical disorders and increased persistence severity of such secondary physical disorders when they occur [7]. Other important indirect costs associated with reduction in quality of life [8], social role functioning [9], and burdens experienced by family members [10]. MDD also has substantial and diverse negative effects on the health and well-being of the children of parents with depression that can be reversed with successful treatment of parental MDD [11].

Estimates from the most recent US Medical Expenditures Panel Surveys suggest that 8% of US adults receive MDD treatment over a 12-month time period, with 87% receiving antidepressant medication (ADM), 23% psychotherapy, and 10% combined ADM-psychotherapy [12]. A number of ADM classes exist, but none is consistently superior to others, resulting in ADM treatment recommendations being based largely on tolerability and safety [13]. A number of evidence-based psychotherapies also exist, with little evidence of differences in effects, but cognitive behavioral therapy (CBT) has the most consistent evidence of effectiveness because it has most often been studied and can most reliably be implemented [14, 15]. Controlled trials comparing ADM to face-to-face CBT find generally comparable aggregate effects [16]. However, controlled trials typically examine only aggregate effects and do not consider the possibility that patients might differ in the treatment that is most helpful to them. The growing amount of research that investigates this possibility of heterogeneity of treatment effects (HTE) finds considerable evidence that HTE exists for MDD treatment [17]. For example, one small trial studying MDD HTE estimated that a clinically significant advantage of either CBT or ADM over the other exists for more than 60% of primary care patients with MDD, suggesting that optimal treatment selection when both CBT and ADM are available would result in a substantial increase in treatment response [18]. Another group of trials randomized patients either to ADM, CBT, or combined ADM-CBT and found that combined treatment had a roughly 50% higher aggregate MDD symptom remission rate than either of the monotherapies [19]. HTE analyses in these studies documented over two dozen consistently significant predictors of HTE across the broad categories of ADM only, CBT-only, and combined treatment, but only a handful of these predictors were included in any single trial and no attempt has ever been made to develop a comprehensive HTE model with all these predictors [20].

Other trials compared guided internet-based CBT (i-CBT) and found aggregate effects generally comparable to those of face-to-face CBT, but at much lower cost [21, 22]. Guided i-CBT is internet-based CBT completed by the patient with a remote guide or coach who communicates with the patient via email, text, or telephone. Coaches also provide elements of remote collaborative care case management [23, 24], such as encouraging ADM adherence, monitoring ADM side effects and treatment response, coordinating with the primary care physician, and facilitating specialty referral. A limitation of these trials, though, was that no HTE analyses were carried out, making it impossible to determine if i-CBT is helpful in all cases or if its value is limited to a subset of patients that can be well-defined before the beginning of treatment [25]. A final relevant group of trials found that self-guided i-CBT had worse aggregate effects than guided i-CBT [25, 26], but significantly better effects than a waiting list control group [27–29]. HTE analyses were not carried out, although the effect of self-guided i-CBT was comparable across levels of baseline MDD symptom severity [30]. Self-guided i-CBT is CBT completed by the user on the internet with computerized feedback but no clinician involvement after an initial orientation meeting.

Six major gaps in evidence exist in the above trials. First, few of them evaluated patient-centered outcomes. Second, the trials that compared ADM only and CBT-only with combined ADM-CBT lack external validity. That is, their results are limited to the small proportion of patients that agreed in advance to be treated either with ADM only, CBT-only, or combined ADM-CBT. This makes it impossible from these trials to evaluate the incremental effect of CBT over ADM only among patients with a strong preference for ADM who would be willing to try CBT in addition to ADM even though they would not be willing to try CBT in the absence of ADM. Trials that add CBT to ADM among patients receiving ADM would be needed to do that [31]. This is what we are doing in our trial. Third, prior trials comparing combined ADM-CBT to ADM only were limited largely to the evaluation of face-to-face CBT, which is an unrealistic option for the great majority of patients with depression due to the limited and declining number of psychotherapists in the population [32] and the fact that only a minority of psychotherapists are fully trained in CBT. Fourth, HTE analyses were limited to only a handful of prescriptive predictors in any single study [33]. Fifth, when more than a handful of prescriptive predictors were considered [18, 34], the sample sizes were too small to generate stable multivariate HTE estimates, leading to a focus on dimensional outcomes and using statistical methods that almost certainly over-fit the data. Sixth, HTE analyses usually used suboptimal analysis methods.

We plan to address all these gaps in the Appalachian Mind Health Initiative (AMHI), a pragmatic trial of the comparative effectiveness of two levels of remote i-CBT added to ADM compared to ADM only. The sample will consist of 3360 patients seeking primary care MDD treatment in West Virginia. AMHI is being carried out by researchers at West Virginia University (WVU) School of Medicine. An evaluation of the incremental benefit of i-CBT is of special importance in West Virginia for several reasons. First, combined ADM-psychotherapy is recommended in some treatment guideline for patients with moderate-severe MDD [35] and comorbidity [36], both of which are elevated in West Virginia due to the state having the 2nd lowest per capita income in the country [37], the highest proportion of residents covered by Medicaid in the country [38], and the highest opioid death rate in the country [39]. West Virginia is also the 2nd most rural state in the country [40]. This confluence of factors results in the proportion of patients with MDD in West Virginia receiving psychotherapy being only about half the national average. This is part of a larger pattern in which West Virginia ranks only 42nd across the 50 states in overall mental health care [41], with the great majority of treatment occurring in primary care settings and consisting of ADM only. Patients who access psychotherapy typically do so only after being on long waiting lists (often 3+ months) and traveling substantial distances to receive treatment. Access to telephone or videoconference psychotherapy is limited. Yet 75% of primary care patients with depression express a desire for psychotherapy either alone (40%) or in combination with ADM (35%) [42]. This mismatch between treatment availability and preference is important because MDD remission increases substantially when patients are not treated with their preferred type of treatment [43–45]. There is thus good reason to believe that providing access to i-CBT will improve MDD treatment outcomes in our trial.

### Objectives {7}

The first objective of AMHI is to evaluate the aggregate incremental effects of combining either best practices guided or self-guided i-CBT to ADM among patients seeking primary care treatment for MDD in West Virginia. The second objective is to determine whether stable predictors can be found of HTE in order to develop a clinical decision support system that generates an individualized treatment rule (ITR) to help patients and clinicians decide whether to add either self-guided or guided i-CBT to ADM primary care treatment of MDD. The ITR will also help identify patients for whom ADM only and combined ADM-i-CBT delivered in primary care both have low probabilities of resulting in MDD remission. A third (exploratory) objective is to use nonexperimental methods to investigate HTE with respect to two major uncontrolled aspects of MDD treatment: type of ADM; and i-CBT versus live psychotherapy (the latter obtained by 12% of primary care MDD patients in West Virginia). We hypothesize that substantial additional HTE will be documented in these nonexperimental analyses and that treatment selection across both randomized and major non-randomized aspects of treatment based on knowledge of this HTE could increase the MDD remission rate significantly.

### Trial design {8}

AMHI will be a three-arm single-blind individually randomized equal allocation controlled pragmatic trial. It will compare aggregate superiority of ADM plus either self-guided or guided i-CBT over ADM only. It will also evaluate the extent to which HTE exists. An extensive baseline internet-based patient self-report questionnaire (SRQ) will be administered prior to randomization. Results will be used to randomize eligible patients across study arms using the finite selection model [46]. As detailed below, i-CBT will typically be completed within 3 months. Brief SRQs will be administered at 2, 4, 8, and 13 weeks to monitor intervention uptake and continued engagement. SRQs at 16 weeks will be used to determine remission (the primary outcome) and various aspects of treatment response (secondary outcomes). Subsequent SRQs at 26, 39, and 52 weeks will be used to monitor maintenance of remission and longer-term outcomes among patients that did not remit previously. Phone calls will be used to obtain patient-reported outcomes data when the internet-based SRQs are not completed. Telephone assessors will be blinded. Informed consent will be obtained to merge electronic medical records (EMR) with self-report data to enrich the dataset and adjust for SRQ loss to follow-up.

## Methods: participants, interventions, and outcomes

### Study setting {9}

Primary care facilities from three established networks throughout the state of West Virginia are being recruited to participate in AMHI: The West Virginia Practice Based Research Network (WVPBRN); the West Virginia Primary Care Association (WVPCA) network; and the anticipated inclusion of the Veterans Health Administration (VHA) system. The WVPBRN is a group of primary care practices with the majority considered Federally Qualified Health Centers. The WVPCA is a private, non-profit membership association that represents West Virginia safety-net health care providers. The WVPCA is also the federally designated primary care association for the state and is the link between federal, state, and local entities providing healthcare for 25% of the state’s residents. The VHA is the largest health care system in the nation and has a strong presence in West Virginia [47] due to the fact that West Virginia has a much higher concentration of Veterans than most states [48]. Recruitment of sites is still under way. and the final set of participating sites is to be determined. The target is to recruit 50 practices with a total of 100 participating clinicians.

### Eligibility criteria {10}

Table 1 lists inclusion and exclusion criteria. The criteria were chosen to recruit a broadly representative sample of primary care patients under treatment for MDD in primary care settings throughout the state. Patients seeking primary care treatment for MDD will need to be in treatment for depression the first time in the past 6 months to be eligible, as the focus is on new episodes of treatment. They will need to be adults (aged 18+) and have a level of severity that does not require hospitalization. They will differ widely in severity, course of illness, and comorbidities. Given that the trial will focus on patient-reported assessments, patients will be required to be literate in English, have access to a telephone, and either have access to a smart phone or computer or be willing to travel to access a tablet computer at their doctor’s office for periodic SRQs. In addition to the exclusion of patients with treatment of MDD within the prior 6 months or current need for inpatient treatment, patients will be excluded if they have an impairment that would interfere with completing the study tasks (i.e., hearing or vision loss), a history of either bipolar disorder or psychosis (based on either EMRs or baseline self-report), or acute serious suicide risk based on self-report of suicide ideation with active suicide intent.

**Table 1:** Appalachian Mind Health Initiative (AMHI) inclusion and exclusion criteria

**Inclusion criteria**	
Participants must meet the following criteria:	
1. Seeking MDD treatment for the first time in the past 6 months (i.e., the beginning of a first or new course of treatment)	
2. Aged 18 years or older	
3. Appropriate for outpatient treatment (i.e., do not require inpatient psychiatric treatment)	
4. Literate in English	
5. Access to a telephone	
6. Access either to a smartphone or computer or willing to travel to access a device at the doctor’s office	
**Exclusion criteria**	
A person is not eligible if any of the following apply:	
1. Treatment of MDD within the past 6 months	
2. Required inpatient psychiatric treatment at the time of the current MDD diagnosis	
3. History of hearing, vision, or cognitive impairment that would interfere with participation	
4. History of either bipolar disorder or non-affective psychosis either in medical records or self-report, or as indicated by treatment with a mood stabilizer or antipsychotic medication	
5. Acute serious suicide risk	

### Who will take informed consent? {26a}

Potential study participants will be identified either at the clinics on the day of initial treatment contact or upon review of records by clinic staff at the end of the workday. If identified while at the clinics, a study fact brochure will be provided to the patient after obtaining preliminary consent for study staff to call them at home to explain the study in more detail. The brochure will contain the study website URL and an 800 number for additional questions or to opt out prior to receiving a call from a study staff member. Potential participants missed during the day will be determined by clinic staff based on review of records at the end of each workday. A letter will be sent to these patients along with the study fact brochure explaining the study and informing these patients that a study staff member will call to explain the study. The letter will also include the study 800 number for patients that want to opt out. Contact information for potential participants will then be sent to the study team using a secure web application in the patient’s electronic medical record. This information will be password protected and access will be limited to those with a need to know. Trained WVU research staff will then contact consented patients within 24 h of their clinic visit to explain the study, answer questions, and obtain verbal informed consent. All research staff involved in consenting participants will complete the Collaborative Institutional Training Initiative training. Only those who have satisfactorily demonstrated the ability to follow consenting protocols and procedures will be approved to consent participants. Electronic informed consent will then be obtained prior to the beginning of the baseline internet-based patient SRQ.

## Interventions

### Explanation for the choice of comparators {6b}

As noted in the introduction, the great majority of patients with MDD prefer psychotherapy either instead of or in addition to ADM [42]. Yet only a small minority (12% in West Virginia; 23% in the total USA) of MDD patients receive psychotherapy [12]. This is a problem both because MDD treatment preference is a strong predictor of treatment response [43–45] and because combined ADM-psychotherapy is known to be more effective than ADM only, especially among patients with moderate-severe depression and psychiatric comorbidity [19]. Face-to-face CBT, the most widely studied evidence-based psychotherapy, is not a realistic option for most MDD patients, especially in a poor rural state like West Virginia, making it important to know whether less expensive and easily scalable i-CBT would improve treatment outcomes if added to ADM compared to ADM only. We consequently decided to focus on a three-arm trial comparing treatment outcomes associated with ADM only versus ADM combined with either self-guided or guided i-CBT. Following a review, the specific version of i-CBT we selected is SilverCloud [49], a leading evidence-based digital i-CBT program. SilverCloud was selected based on its extensive evidence base [50–58] and the fact that it can be delivered in either self-guided or guided forms.

### Intervention description {11a}

SilverCloud is a transdiagnostic guided i-CBT platform with 30 programs that can be tailored to the specific needs of users. Programs can be repeated if the user (or, in the case of the guided version, the coach) feels that this would be useful [55]. All programs are available 24/7. Participants in the trial assigned to i-CBT (either self-guided or guided) will all receive the SilverCloud program designed for patients with depression, which is described in Tables 2 and 3. This program is designed to relieve symptoms of depression by teaching more flexible ways of thinking, increasing awareness and understanding of emotions, and increasing activity and motivation in daily life. The program consists of 8 modules, each taking 45–60 min to complete. Users are recommended to complete one module per week and to break up each module into 3–4 sessions of 10–20 min each. In the case of the guided version, coaches provide asynchronous post-session feedback on the work patients have completed on the platform and provide personalized recommendations of content for the users. Coaches can also suggest that users revisit some sections within the module or prior modules within the week based on open-ended text provided by users, whereas this kind of tailoring relies on user selections from menus provided in the self-guided version of the program in addition to weekly email messages sent through the platform for up to 10 weeks. Coaches will be BA-level graduates of behavioral health programs who have been trained in the SilverCloud platform and in how to deliver feedback. In addition to the core depression program, patients can be provided with unlockable modules depending on concomitant issues and difficulties they may be experiencing, such as with sleep, self-esteem, and communication.

**Table 2:** SilverCloud Space from Depression i-CBT 8-module overview

The following modules will be released to all participants assigned to the i-CBT arms	
Session 1: Getting Started: This module introduces the user to cognitive behavioral therapy and explores how it can help the user to understand what is going on inside them and make changes to feel better. It also introduces the user to two of the key tools in the program—the Mood Monitor and the CBT Cycle.	
Session 2: Understanding Depression: This module introduces the user to the cycle of depression and the emotional, cognitive, physical, and behavioral aspects of depression. The user is also provided with activities to enable them to reflect on and understand their situation.	
Session 3: Noticing Feelings: This module focuses on emotions and physical sensations. The aim of this module is to help the user to understand and identify their emotions and their association with low mood. This module also addresses the physical sensations that are associated with depression, and the importance of considering the impact of lifestyle choices on low mood. The user can begin to build their own CBT cycles and track the impact of their lifestyle choices on their low mood in this module.	
Session 4: Boosting Behavior: This module focuses on one of the core issues of depression—inactivity and a lack of motivation. The user is introduced to the cycle of inactivity and its role in maintaining depression. This module helps to user to identify ways to motivate themselves to engage in pleasurable activities and activities that provide a sense of achievement. The user also learns about practical strategies to tackle the unpleasant physical feelings associated with depression.	
Session 5: Spotting Thoughts: This module focuses on the “thoughts” component of the CBT cycle and introduces the user to negative thinking and its impact on mood. The user is introduced to a number of thinking traps and is encouraged to try and identify their negative or unhelpful thoughts. The activities allow the user to continue to build their CBT cycles.	
Session 6: Challenging Thoughts: This module focuses on taking action against negative thoughts. The user is introduced to “hot thoughts” and their impact on low mood. This module helps the user to learn techniques to tackle the various thinking traps that are common in depression and to identify alternative ways of thinking. This module also introduces the user to coping thoughts and helpful self-talk thoughts.	
Session 7: Core Beliefs: Many people with depression struggle with the “thoughts” component of the CBT cycle. Although they may be able to identify unhelpful thoughts and thinking traps, they may struggle to identify alternatives or generate coping thoughts. The *Core Beliefs* module was developed to specifically target the deeply held core beliefs that are the underlying root of these unhelpful thoughts and keep the cycle of depression and low mood going. This module helps the user to identify healthy and unhealthy core beliefs and teaches them strategies to challenge core beliefs and generate more balanced alternatives.	
Session 8: Bringing It Altogether: This module prepares the user for coming to the end of the program and focuses on helping them stay well in the future. The user learns about warning signs that their mood is deteriorating and how to plan to ensure that they stay well. This module also highlights the importance of social support and continuing to use the skills and techniques that they have learned to prevent future relapse. The user has the opportunity to review the expectations that they had at the start of the program and can set goals for the future.

**Table 3:** SilverCloud Space from Depression i-CBT modules, topics, goals, and activities

Modules	Topics	Goals	Activities
**1. Getting Started**	• Introduction to CBT model • The CBT Cycle • Personal stories	• Learn about CBT • Introduce the Mood Monitor • Introduce the CBT Cycle • Learn how thoughts, emotions, physical sensations & behaviors affect each other • Connect with the present moment	• Mood Monitor • My CBT Cycles • Staying in the Present (Breathe)
**2. Understanding Depression**	• Psychoeducation • Applying CBT to depression • The cycle of depression • Personal stories	• Improve understanding of depression • Learn about role of thoughts, emotions, physical sensations and behaviors in depression • Reflect on own personal circumstances	• Depression Myths & Facts Quiz • Understanding My Situation • Staying in the Present (Body Scan)
**3. Noticing Feelings**	• Understanding emotions • Managing emotions • Physical sensations & mood • Lifestyle choices • Changing physical sensations to improve mood • Personal stories	• Learn about emotions and role in CBT Cycle • Recognize emotions that are difficult to cope with • Recognize physical sensations • Identify activities to target distressing physical sensations associated with depression • Explore the impact of lifestyle choices on depression and well-being	• Emotions & Your Body Quiz • My CBT Cycles • Mapping Lifestyle Choices • Staying in the Present (Progressive Muscle Relaxation)
**4. Boosting Behavior**	• Psychoeducation • Behavioral traps • Increasing activity level • Helpful/unhelpful supports • Getting motivated • The importance activities • Personal stories	• Learn about the link between mood & behaviors • Improve knowledge of common behavioral traps & how to beat them • Learn tips on how to get motivated during periods of low mood • Recognize the importance of pleasurable activities & achievements in boosting mood	• Mood & Behavior Quiz • Your Backup & Support Network • My Motivational Tips • My Activities • Your Mood & Your Body • Activity Scheduling • Staying in The Present (Mindful Eating)
**5. Spotting Thoughts**	• Automatic thoughts & mood • Thinking traps • Catching unhelpful thoughts • Personal stories	• Learn about the role of thoughts in depression within the CBT Cycle • Recognize negative automatic thoughts • Understand & recognize thinking traps	• Me & My Thoughts Quiz • My CBT Cycles • Staying in the Present (Watching Thoughts)
**6. Challenging Thoughts**	• Hot thoughts • Challenging thoughts • Tackling thinking traps • Coping with situations • Personal stories	• Learn about hot thoughts & how to recognize • Learn to challenge negative thoughts • Learn how to overcome specific thinking traps • Recognize situations where it is necessary to use thoughts to cope	• Your Thinking Style Quiz • My Helpful Thoughts • My CBT Cycles • Staying in the Present (Watching Thoughts)
**7. Core Beliefs**	• What are core beliefs • Where do they come from • Identifying core beliefs • Challenging core beliefs • Balancing core beliefs • Personal stories	• Improve understanding of core beliefs & where they come from • Improve knowledge on how to recognize hot thought themes & underlying core beliefs • Learn to challenge core beliefs by finding evidence • Balance core beliefs using balanced alternatives • Gain insight into experiences of core beliefs	• Core Beliefs Quiz • Core Beliefs: (identifying, challenging, balancing, and strengthening)
**8. Bringing it All Together**	• Finishing up • Warning signs & planning • Social support • Preparing for the future • Preparing for relapse • Personal Stories	• Preparation for coming to the end of the program • Recognize the importance of social support in staying well • Identify warning signs • Planning for staying well • Set goals for the future	• Your Backup and Support Network • Staying Well Plan • Goals • Taking Stock • Staying in the Present (Sounds)

### Criteria for discontinuing or modifying allocated interventions {11b}

Patients randomized into the trial will be monitored via 8 internet-based tracking SRQs at 2, 4, 8, 13, 16, 26, 39, and 52 weeks after randomization regarding important changes in their symptomology and the need for communication with their primary care provider or implementation of a crisis management strategy (i.e., a participant expresses intent to harm themselves or others). Each tracking survey will contain questions assessing suicidality within the past 2 weeks. Participants who report thinking of suicide or death several times a day in some detail with at least some intent of acting on these thoughts will receive a closing statement at the end of their survey encouraging them to contact the National Suicide Prevention Lifeline (1-800-273-TALK) and/or online chat services (via https://suicidepreventionlifeline.org/). We will also provide the participant’s contact information to the National Suicide Prevention Lifeline for outreach, and we will generate a report to the participant’s primary care treatment provider. The Principal Investigator (PI) or designee will then notify the participant’s primary care treatment provider about the incident. The reviewing primary care treatment provider will then determine whether the participant should remain in the study. Other reasons for discontinuation from the study will be a patient’s need for hospitalization as assessed by their primary care treatment provider, clinically significant adverse events not consistent with continuation in the study as determined by the study team or the participant’s primary care treatment provider.

### Strategies to improve adherence to the interventions {11c}

i-CBT users often exhibit low levels of engagement, especially in disadvantaged populations [59]. We will address this problem using several strategies. First, patients assigned to i-CBT will both be (i) sent email messages notifying them of these assignments and (ii) receive a phone call from study staff using motivational interviewing techniques to encourage engagement [60]. Second, the first two brief SRQs, at 2 and 4 weeks after randomization, will be used as additional occasions to monitor intervention engagement. Patients that either fail to respond or (in the case of the i-CBT arms) report that they have not yet started the intervention will receive additional motivational interviewing contacts to encourage engagement with the assessments and (in the i-CBT arms) the interventions. Consistent with previous research [61, 62], we anticipate that these frequent contacts will increase engagement. Third, we will attempt to build on a recent process analyses of meta-data from over 50,000 SilverCloud users, which identified five early longitudinal user engagement profiles that predict treatment response [63]. To the extent possible, we will score these clusters for each patient assigned to SilverCloud and use the profile scores to target the subset of patients identified as non-engagers in weeks 3–4 of the trial for additional motivational outreach email messages.

### Relevant concomitant care permitted or prohibited during the trial {11d}

Eligible patients will be treated for major depression by their primary care physician. We will not control type or dose of ADM prescribed, but we will track dose, titration, augmentation, and switching through EMRs. Some patients will also be treated with other psychotropic medications for comorbid conditions, such as anxiolytics for comorbid anxiety disorders, stimulants for ADHD, or addiction medications for comorbid substance use disorders, and these will be allowed. However, patients treated with anti-mania medications or antipsychotics will be ineligible for the trial due to the exclusion of patients with a history of bipolar disorder or psychosis.

### Provision for post-trial care {30}

Access to SilverCloud will continue for 12 months after randomization for patients randomized to the two i-CBT arms.

### Outcomes {12}

The primary outcome will be MDD remission at 16 weeks as defined by a revised version of the composite patient-centered Remission from Depression Questionnaire (RDQ) [64, 65]. The RDQ is a patient self-report scale that assesses the 7 dimensions found in extensive research to be the ones most important to patient-centered definitions of MDD recovery: [66–68] remission from depressive and non-depressive (e.g., anxiety) symptoms, positive mental health, coping ability, productive and social role functioning, life satisfaction, and global sense of well-being. The RDQ is the best validated patient-reported outcome measure of these 7 dimensions. It has excellent psychometric properties, is as sensitive to change as symptom-based scales, but captures additional information [64, 69]. Importantly, patients consistently say that the RDQ represents their treatment goals more than standard symptom scales [70]. Based on evidence of a strong second-order factor across the 7 RDQ dimensions [64], an aggregate score can also be derived. Our primary outcome will be a dichotomous definition of remission based on this aggregate score at 16 weeks and of maintenance of remission at 26, 39, and 52 weeks. The cutoff for this designation in the RDQ was calibrated by the RDQ developers to balance false positives and false negatives in using composite RDQ scores to predict patient reports of “being completely back to normal.” This calibration exercise will be replicated in AMHI to guarantee the internal validity of results. In addition, the depression symptom severity scale used in AMHI will be different from the one in the RDQ: the Quick Inventory of Depressive Symptomatology Self-Report (QIDS-SR) [71]. The QIDS-SR will be used because of its strong association with the gold standard Hamilton Rating Scale for Depression (HRSD) [72] and the existence of a validated crosswalk between the QIDS-SR and HRSD [73]. The focus on a dichotomous measure of remission as the primary outcome is based on evidence that MDD remission is critical for reducing recurrence, leading treatment guidelines to call for MDD to be treated to remission [35, 74]. The 7 dimensional RDQ and QIDS-SR scores will be secondary outcomes. Given the prominence of substance use disorders in West Virginia [75] and the high comorbidity of MDD with these disorders in the population [76], we will also include a substance use disorder symptom scale [77] as a secondary outcome. Anxiety comorbidity, while also of interest, is already addressed in one of the RDQ dimensions. Finally, a measure of ADM treatment compliance based on EMR data, measures of treatment engagement based on both self-reports and administrative records [78], and a patient-reported measure of shared decision-making [79] will be additional secondary outcomes.

### Participant timeline {13}

**Fig. 1:**
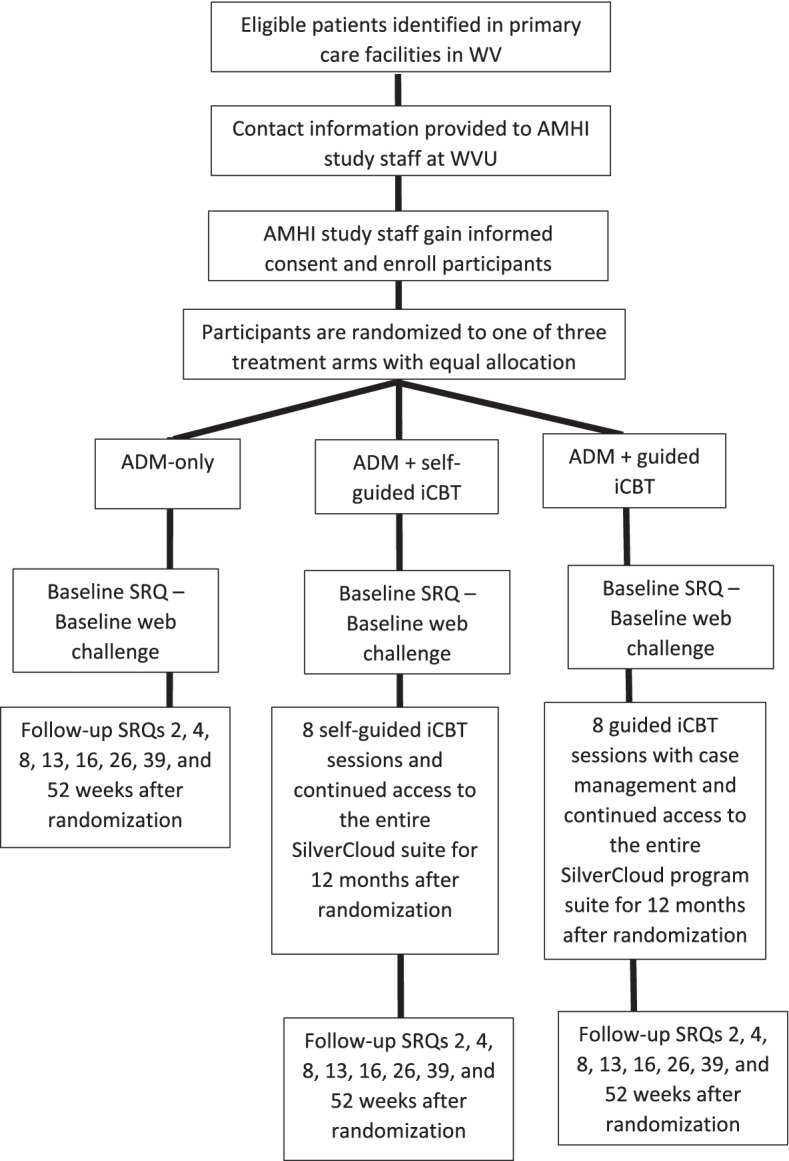
Participant timeline

The participant timeline is outlined in Fig. 1. Potential study participants identified at participating clinics during their initial visit will be informed of the study by their treatment provider. Additionally, they will be provided with a study fact brochure, which contains highlighted study information as well as the study website URL and an 800 number for additional questions. Participating clinics will then gain permission from their potentially eligible participants to provide AMHI study staff with their contact information for enrollment. Potentially eligible patients who were not informed of the study during their initial treatment visit will be sent a letter with the signature of their treatment provider along with the study fact brochure explaining the study and informing these patients that a study staff member will call to further explain the study and discuss possible enrollment. The letter will also include the study 800 number for patients that want more information or to opt out. AMHI study staff at WVU will then contact, gain informed consent, and subsequently enroll each participant into the study. Upon receipt of informed consent, potentially eligible participants will be emailed a link to complete the baseline SRQ and an online web challenge to evaluate cognitive performance. Given the nature of eligibility criteria, a final decision about eligibility will be made only after completion of the baseline SRQ and online web challenge. Eligible participants will then be randomized to one of the three treatment arms. The finite selection model will be used to increase balance in baseline covariates across treatment arms [46]. Email will be used to notify patients of these assignments. Participants in the i-CBT arms will then receive a phone call from study staff using motivational interviewing techniques to encourage engagement with the intervention [55]. Regardless of the arm the participant is randomized to, subsequent SRQs will then be administered at 2, 4, 8, 13, 16, 26, 39, and 52 weeks post randomization. Participants that fail to complete SRQs will receive a set of reminder emails encouraging completion. Additionally, participants that fail to complete SRQs in weeks 2, 16, 26, and 52 will receive phone calls using motivational interviewing techniques to complete SRQs.

### Sample size {14}

We will enroll a target enrollment sample of 3360 patients who complete the baseline SRQ and are randomized across the three treatment arms (i.e., 1120 per arm). The sample was powered to detect predictors of HTE that would increase the proportional remission rate by 20% by optimally assigning individuals as opposed to randomly assigning them into three treatment groups of equal size. A much smaller sample size would be adequate to address the first study objective of evaluating the significance of differences in aggregate remission rates across the 3 treatment arms. Meta-analyses of previous trials show that the MDD symptom remission rate averages about 25% among MDD patients randomized to ADM only and 37.5% among patients randomized to combined ADM-psychotherapy [20]. Although there were no meta-analyses of remission rates for self-guided i-CBT at the time AMHI was planned, other meta-analyses showed that effects of self-guided i-CBT were significantly better than controls [30] but worse than guided i-CBT [25, 26]. We consequently assumed, for purposes of power calculations, that the patient-centered remission rate for ADM plus self-guided i-CBT in AMHI would be 31%, which is roughly midway between the extremes. Power to detect each of the 6% differences between the extremes and the middle category using a 0.05-level 1-sided test and assuming 30% loss to follow-up (which is typical of i-CBT trials) is .86, whereas power to detect the 12.5% difference between the two extremes using the same specifications is .99.

Evaluating power to detect HTE is more complex and requires simulation. We did this by beginning with data on the observed distributions and exogenous associations among the baseline predictors in the STAR*D trial [80] and assumed that each patient was randomized across three treatment arms. We then specified a series of relatively complex nonlinear-interactive multivariate classification models for associations of these predictors with MDD remission, assuming plausible prognostic and prescriptive coefficient values that generated population distributions with the same aggregate outcome prevalence as assumed above [81]. We then modified the prescriptive coefficients to retain the aggregate remission rates while embedding HTE in the population that would result in a 20% proportional increase in the aggregate remission rate under randomization with equal allocation (i.e., from 31% to 1.2 × 31% = 37.5%). We then drew 500 pseudo-samples of different sizes from this simulated population and applied our HTE analysis method (see below) to estimate HTE in these sample data. We assumed that all the significant prognostic and prescriptive predictors were measured in the samples in addition to 20 noise predictors. Power was calculated as the proportion of replicates in which the lower bound of the 95% CI of the HTE estimate was greater than 0.0%. We also calculated proportional regret (i.e., downward bias in the estimated versus true proportional increase in remission under optimization vs. randomization). The sample size was set to yield power greater than .80 and proportional regret less than .20.

### Recruitment {15}

We anticipate a 24-month recruitment period. As outlined in Table 4, initial eligibility will be determined during the patient’s clinic visit. Potentially eligible participants will be provided with a study fact brochure, which contains highlighted study information as well as the study website URL and an 800 number for additional questions. Participating clinics will then gain permission from eligible participants to provide AMHI study staff with their contact information. Eligible participants who were not informed (i.e., missed in recruitment) will be sent a letter explaining the study along with a study fact brochure under the signature of their treating clinician. This letter will contain the study 800 number for patients who would prefer to opt out. Patients who do not opt out will receive a telephone call from AMHI study staff to reassess eligibility, review material in the study fact brochure, and answer patient questions before seeking electronic informed consent to participate in the study. Patients who prefer to physically sign the informed consent will be mailed two hard copies (one for their records) along with a pre-addressed pre-stamped return envelope.

**Table 4:** Participant recruitment

**I. Clinic pre-recruitment**	
A. The primary care physician will determine patient eligibility during the clinical appointment and provide the potential participant with a study fact brochure that describes the study and provides an 800 number for questions. Physicians will share the name and phone number of eligible patients with study staff based on patient permission.	
B. Participating clinical facilities will look through charts daily to identify those patients that met inclusion criteria but were not informed of the study by their provider. A cover letter about the study will then be mailed to these patients under the signature of the provider along with the study fact brochure.	
**II. Telephone recruitment**	
A. Patients who do not opt out in Phase 1 A will receive a telephone call within 24 h from the study staff.	
B. Patients who are identified in Phase 1 B will be provided in the letter with an opt-out number to call if they do not want to be contacted by study staff. Staff will attempt to contact patients that do not call to opt out within 72 h.	
C. Once study staff make telephone contact with the participant on the phone:	
1. Study staff will assess eligibility, review the content of the study fact brochure, and answer questions.	
2. Study staff will seek verbal informed consent and contact information including email address and preference for email versus text.	
3. If a participant prefers to physically sign the informed consent script, two hard copies will be mailed to the participant with a pre-stamped pre-addressed return envelope with instruction to keep one copy and mail back the second signed copy.	

## Assignment of interventions: allocation

### Sequence generation {16a}

Baseline SRQ results will be used to stratify randomization of eligible patients across study arms. This will be done initially using the 3-way cross-classification of depression symptom severity, chronicity, and comorbidity based on a SAS macro that will be run daily by the WVU study data manager until 100 participants are assigned per arm. The finite selection model [46] will be used subsequently to randomly assign patients and balance on a larger set of variables, again with the WVU study data manager implementing the procedure daily.

### Implementation {16c}

Initial stratification by the 3-way cross-classification of depression symptom severity, chronicity, and comorbidity will be implemented by a SAS macro that is independent of the WVU data manager that implements the assignment. The subsequent stratification based on the finite selection model will also be implemented by computer, again independent of the WVU data manager that implements the assignment.

## Assignment of interventions: blinding

### Who will be blinded {17a}

Clinical staff at primary care facilities and telephone recruiters will be blinded at the time of recruitment to treatment assignment, which will take place after recruitment is complete. Telephone interviewers that obtain self-report information from participants who did not complete their SRQ will also be blinded to treatment arm.

### Procedure for unblinding if needed {17b}

If concerning symptomology emerges as defined in the Data Safety and Monitoring plan, the participant will be flagged and reported to the PI for review. If a participant’s SRQ indicates acute serious suicide risk, a closing statement in the SRQ will encourage the participant to contact the National Suicide Prevention Lifeline and will inform the participant that both their treating physician and the National Suicide Prevention Lifeline are being informed of their suicidality.

## Data collection and management

### Plans for assessment and collection of outcomes {18a}

Assessments will be carried out with patient-reported SRQs augmented by EMRs that will provide information about relevant baseline information (e.g., information about treatment history prior to the intervention that might be relevant in predicting outcomes or HTE) as well as treatment information over the course of the trial involving both the ADM (type, dose, titration, switching, augmentation) and other treatments that might be relevant to outcome assignment among patients lost to follow-up in the SRQ assessments (e.g., psychiatric hospitalizations, emergency department visits for mental health crises, suicide attempts, suicide deaths, other deaths by external cause). Additionally, assessments will be gathered via smartphone with consenting participants capturing ecological momentary assessments as well as sensor data from the smartphone app mindLAMP [82, 83] that can be used to derive estimations for daily physical activity, sedentary activity, sleep duration, screen time exposure, and social behaviors. Participants will be asked to download an Apple or Android version of mindLAMP onto their personal smartphones for these measures.

The SRQs will be collected remotely by a health survey firm that has extensive experience implementing mixed-mode web-phone surveys. Computerized procedures will be used to automate skip logic, flag missing values for completion, disallow out-of-range and inconsistent responses, and discourage superficial responses. This survey firm will also carry out the telephone interviews with SRQ nonrespondents. Each SRQ will be sent by email. Reminders to initial nonrespondents will be sent 3 and 6 days later. Options will be provided for participants who want to break up an SRQ for completion over multiple sessions. Telephone follow-up calls and interviews with initial SRQ nonrespondents will be carried out by the survey firm in conjunction with the SRQs at 2, 16, 26, and 52 weeks. The survey firm will also maintain an 800 number for technical assistance.

As noted above, more than two dozen consistently significant baseline patient-reported predictors of MDD HTE have been documented in the literature [33]. These are outlined in Table 5. We developed a baseline self-report SRQ to assess these predictors by carrying out a systematic literature review of the best available short-form patient-reported measures of each construct. We also worked with statisticians and psychometricians to develop optimal short-form scales in secondary analyses to create new short-form versions of existing scales when none already existed [84–86]. We made extensive use of intelligent skip logic in designing the baseline SRQ to shorten the assessment once scale scores in a relevant range could be inferred from partial responses.

**Table 5:** Baseline predictors

Baseline constructs	Predictors
Demographics	Age [87], gender [87], educational attainment employment status [87], occupation/industry [88], marital/relationship status [87], number of children [87]
Depression history and features	Depression symptom severity [64, 89]; Melancholic features [90–92]; Mixed features [91, 93, 94]; Anhedonia [95, 96]; Atypical depression [64, 89, 90, 97], Endogenous depression [98–100]; History and persistence [98–100]; Suicidality [101–103]
Other comorbid disorders/symptoms	Anxiety [64, 91, 99]; Panic [99]; Anger [98]; Irritability [64]; Dissociative symptoms [91, 99]; PTSD [104, 105]; Substance use/abuse [87, 106]; Psychotic symptoms (exclusionary); Bipolar disorder (exclusionary); Personality disorders [1, 107, 108]; Other comorbid disorders [98, 109, 110]; Somatization/somatic anxiety [91, 111–114]; Sleep problems [115]; Pain [98, 116, 117]; Role functioning/impairment [118]
Stress and adversity	High current stress [98]; Stressful life events [98, 119, 120]; Childhood trauma and maltreatment [121–124]; Parental bonding [125]; History of Traumatic Brain Injury [98]; Social media use [126]; Social support [99, 127]; Perceived belongingness and burdensomeness [128]; Loneliness [129]; Religiosity [98–130]; Masculinity norms [131–133]
Personality traits and temperament	Alexithymia [134]; Attentional control [135]; Attachment style [136]; Emotional regulation [137]; Self-blame [138]; Positive reappraisal [138]; Coping ability [64]; Life satisfaction [64]; General sense of well-being [64]; Positive sense of mental health [64]; Posttraumatic growth [139]; Perceived self-efficacy/control [140]; Resilience [141–143]; Stress reactivity [144]; Hopelessness [145]; Rumination [146]; Negative affect/neuroticism [84, 147–156]; Emotionality [149, 156]; Openness to experience [84, 98, 157]; Extraversion [84, 147, 148, 152, 153, 158]; Conscientiousness [84, 149, 152, 157]; Mastery [159]; Self-esteem [160]; Problem-solving ability [161]; Cyclothymic temperament [147, 148, 153, 162]; Hyperthymic temperament [147, 148]
Treatment engagement and related constructs	Healthcare utilization [94]; Continuity of care [163]; Health literacy [164]; eHealth literacy [165]; Treatment history [94]; Adherence [166–168]; Therapeutic alliance [169]; Patient-provider communication [170, 171]; Shared decision-making [79]; Patient preferences [172]; Acceptability and willingness [173]; Expected medication side effects [174]; Treatment expectancies [175]
Other	Concentration/decision making [176]

We also noted above that the primary outcome in AMHI will be remission defined by a modified version of the composite RDQ [64] that substitutes the QIDS-SR [71] for the RDQ depression symptom severity scale. Secondary outcomes in addition to the component dimensional RDQ and QIDS-SR scores will include a substance use disorder symptom scale [77], a measure of ADM treatment compliance, measures of patient engagement in treatment based on both self-reports and administrative records [78], and a patient-reported measure of shared decision-making [79], all of which are based on widely-used self-report scales that have good psychometric characteristics as shown in Table 6.

**Table 6:** Self-report questionnaire (SRQ) outcomes

	Assessment timepoints							
	2 weeks	4 weeks	8 weeks	13 weeks	16 weeks	26 weeks	39 weeks	52 weeks
**Primary outcome**								
Dichotomous definition of remission from depression^a^					X	X	X	X
**Secondary outcomes**								
Dimensional remission from depression scores^a^					X	X	X	X
Substance use disorder symptoms^b^					X	X	X	X
Treatment compliance^c^	X	X	X	X	X	X	X	X
Treatment engagement^d^	X	X	X	X	X	X	X	X
Shared decision making^e^								X
Perception of recovery/remission^f^	X	X	X	X	X	X	X	X
Treatment satisfaction^f^	X	X	X	X	X	X	X	X
**Other measures**								
Current stressors^g^	X	X	X	X	X	X	X	X
Current treatment^h^	X	X	X	X	X	X	X	X
Medication side effects^i^	X	X	X	X	X	X	X	X

^a^Operationalized with questions from the Remission from Depression Questionnaire (RDQ) [64], 16-Item Quick Inventory of Depressive Symptomatology Self-Report Scale (QIDS-SR) [89], full Inventory of Depressive Symptomatology Self-Report Scale (IDS-SR) [90], Composite International Diagnostic Interview (CIDI) [94], DSM-5 Anxious Distress Specifier and Melancholic Features Specifier for Depressive Disorders [91], and the Sheehan Disability Scale (SDS) [118]

^b^The 7-question PROMIS Alcohol/Substance Use Short Form - 7a scale [87, 106]

^c^Adapted two questions from the Brief Adherence Rating Scale (BARS) [177] to assess medication and psychotherapy treatment compliance [78]

^d^i-CBT engagement will be monitored by analyzing SilverCloud meta-data

^e^The 3-item CollaboRATE scale [79]

^f^Created questions to measure patient perceived remission [178, 179]; and treatment satisfaction [180]

^g^Questions taken from Army STARRS Survey [98]

^h^Items developed for AMHI Study

^i^The Frequency, Intensity, and Burden of Side Effects Ratings (FISBER) scale [174]

### Plans to promote participant retention and complete follow-up {18b}

It will be made clear to participants at the onset of the recruitment process that they will be expected to complete a series of 10 study tasks (baseline SRQ and online challenge tasks to evaluate cognitive performance; and follow-up SRQs at 2, 4, 8, 13, 16, 26, 39, and 52 weeks). We will use a citizen-scientist model to appeal to participants to complete as many of these assessments as possible, emphasizing the special importance of the 16-week and 52-week assessments. Participants will be paid for their time completing all assessments, including $50 for the baseline SRQ and $50 for the cognitive challenge task, $50 for the 52-week SRQ, and $20 for each of the other SRQs. Reminder emails and texts will be used to increase response. In the case of the 2-, 16-, 26-, and 52-week assessments, we will also use telephone reminder calls both to encourage SRQ completion and to collect this information via telephone interview when we cannot do so by SRQ.

### Data management {19}

The WVU study team will maintain a master dataset for all patients who were referred to the project for recruitment along with their dispositions (i.e., withdrew from participation before or after completing the baseline SRQ or after initiating the intervention, were judged ineligible before or after completing the baseline SRQ, were terminated from the study after initiating the intervention, continued throughout the study). Research ID numbers will be assigned separately to this disposition file and to a file containing all personally identifying information (PII) but the PII will not be linked directly to the disposition file. The WVU team will also create a master EMR data file that contains research ID numbers in addition to EMR data for all participants but contains no PII. The master EMR data file will contain information abstracted directly from the EMRs of participants who provide consent to share this information. AMR data will be managed as a SAS data file stored on a HIPPA compliant server maintained by WVU. The survey firm will maintain a separate consolidated master SRQ data file for each participant that contains the same research ID numbers as those used by the WVU team but contains no PII. A separate secure datafile will be maintained by the survey firm that contains only the research ID and the PII of each participant. The de-identified master EMR data file and the master SRQ data file will be merged for data processing by the WVU study team. All data analyses will be carried out with this de-identified consolidated data file jointly by the WVU and Harvard Medical School (HMS) collaborators. Access to participant data will be restricted to members of the study team listed on the IRB-approved protocol.

### Confidentiality {27}

Access to PII will be restricted to study staff identified on the IRB-approved protocol. When appropriate, information on risk for harm to self or others or significant worsening of symptomology will be reported to the patient’s primary care clinician and, in the case of acute serious suicidality, to the National Suicide Prevention Lifeline. A Certificate of Confidentiality has been obtained for this study from National Institutes of Health.

## Statistical methods

### Statistical methods for primary and secondary outcomes {20a}

The WVU analysis team will construct summary EMR variables for patients and treatment providers behind the WVU firewall and transfer these data to the survey firm via secure file transfer for linkage with the SRQ dataset behind the survey firm’s firewall. These data will be de-identified before returning to WVU and HMS for analysis. Objective 1 analyses will evaluate aggregate differences across the treatment arms. We will use logistic regression to estimate binary outcomes and report adjusted prevalence ratios with design-adjusted 95% confidence intervals (CIs). We will calculate number needed to treat (NNT) for each comparison. Generalized linear models will be used to estimate effects on continuous outcomes, making use of standard visual diagnostics to choose appropriate link functions and error structures [181]. We will report adjusted mean differences with design-adjusted 95% CIs [182].

Objective 2 analyses will evaluate HTE across the three randomized treatment arms using a special case of the super learner (SL) algorithm [183], an ensemble machine learning approach that uses cross-validation (CV) to select a weighted combination of predicted outcome scores across a collection of candidate algorithms that yields an optimal weighted combination according to a pre-specified criterion that performs at least as well as the best component algorithm. The candidate algorithms in SL can either be parametric or flexible machine learning algorithms, making SL less prone to model misspecification than traditional parametric approaches. The guarantee that SL performs at least as well as the best candidate algorithm allows a rich library of parametric and flexible candidate algorithms to be included.

In the conventional approach to estimating HTE, a model with main effects and interactions between prescriptive predictors and dummy variables for treatment indicators is estimated. Predicted values based on this model are then used to estimate the expected individual-level outcome conditional on the values of the prescriptive predictors for each patient in each treatment condition (e.g., the estimated outcomes of patient *p* under treatment arm *a*, arm *b*, and arm *c*). An estimate of the optimal treatment strategy for patient *p* is then obtained by comparing predicted values of the outcome across all treatment arms. It is important to appreciate that the accuracy of this approach requires correct specification of both the (possibly nonlinear) main effects and the (possibly complex nonlinear and higher-order) interaction terms. SL has two advantages over this conventional approach [184]. First, it requires only correct specification of the interactions. It does not require correct specification of main effects, as it directly estimates contrasts that allow the correct specification of the main effects to be circumvented. Second, unlike earlier approaches to estimating HTE that share this desirable feature [185, 186], SL uses a flexible set of component machine learning algorithms that maximize chances of capturing complex nonlinear and higher-order interactions correctly.

Objective 3 (exploratory) will examine two aspects of treatment that were not randomized: treatment with one of three broad types of ADM (SSRIs, SNRIs, bupropion) for which there is some evidence of HTE, and live psychotherapy combined with ADM rather than i-CBT with ADM. As noted earlier, 12% of West Virginia primary care MDD patients currently receive live psychotherapy. In these analyses, we will estimate a model to predict selection into each nonrandomized type of treatment as a function of baseline covariates to determine if these uncontrolled aspects of treatment are nonrandom with respect to baseline predictors. We will balance on these baseline covariates before estimating the SL model to evaluate the aggregate effects of these aspects of treatment as well as baseline predictors of HTE with respect to these aspects of treatment.

To quantify the potential value of developing a precision treatment rule for i-CBT, we will use an approach that is roughly equivalent to the calculation of NNT in aggregate analyses of treatment effects. Specifically, we will use a cross-validated targeted minimum loss-based estimator (CV-TMLE) [187] of the attained improvement of the mean outcome under a treatment selection scheme that always selects the treatment option with the best predicted outcome compared to the mean outcome under balanced randomization. This CV-TMLE yields an estimator of the attained improvement with minimal bias because it uses CV to separate the estimation of the optimal treatment strategy from the assessment of the estimated strategy’s performance and also by allowing for the incorporation of flexible estimation approaches for the regressions and conditional probabilities needed to define the attained improvement. Given that we will be evaluating the effects of expanding of treatment options rather than deciding between two alternative options (i.e., adding i-CBT to ADM rather than choosing between i-CBT and ADM as alternative monotherapies), we will evaluate a range of decision margins; that is, the expected aggregate effects on overall remission rates associated with patient decisions about adding i-CBT to ADM when individual-level increases in predicted probability of remission are in a given range. In addition, we will quantify the uncertainty in aggregate estimates in CIs.

### Interim analyses {21b}

Interim analyses will be conducted only to assess patterns and predictors of attrition for purposes of improving the targeting of motivational interviewing contacts to improve intervention uptake and continued engagement. Ongoing data monitoring will also be used if requested by the Data Safety Monitoring Board to facilitate recommending changes to study activities. Attending clinicians will be notified when reports of suicide thoughts or behaviors are reported and will inform study staff if there is a recommendation to discontinue study activities. Participants who report acute suicidal thoughts or behaviors will be flagged for follow-up. The study team will inform the participant’s treatment provider when there is a patient-reported suicide attempt or ideation with a plan and intent.

### Methods for additional analyses (e.g., subgroup analyses) {20b}

As noted above, objective 2 is to carry out subgroup analyses that evaluate the significance of HTE and attempt to develop an ITR to make treatment assignments under balanced allocation that optimize the aggregate remission rate.

### Methods in analysis to handle protocol non-adherence and any statistical methods to handle missing data {20c}

Objective 1 analyses will be intent-to-treat [188] analyses using inverse probability weights (IPW) to deal with loss to follow-up [189]. For each time *t* (weeks 2, 4, 8, 13, 16, 26, 39, 52), we will compute the probability of a participant in the study at time *t* remaining in the study up through time *t+1* conditional on information collected as of time *t*. We will use flexible, nonparametric estimation methods with variable selection for confounder control [190]. The IPW as of time *t+1* will be based on the product of these conditional probabilities up through *t+1*. The treatment-specific mean outcome will be estimated using these weights for the subjects whose outcomes were observed at *t+1*. Under a coarsening at random assumption, this estimator converges with increasing sample size to the treatment-arm-specific mean outcome that would have been observed had all subjects remained in the study up through *t+1* [191]. When data are missing, we will provide thorough summaries of reasons for missingness, proportions of missing data, and test for differences in the characteristics of participants with and without missing data and we will describe these and the implications of missing data for interpretation when we report the trial’s results. As our IPW approach to missing outcomes data will be based on a missing-at-random assumption, sensitivity analysis will be carried out based on the weaker missing-not-at-random assumption using pattern mixture modeling [192] in a generalized mixed model framework [193]. The predictors of success in obtaining outcome data at the time we evaluate remission (13 weeks after baseline) and maintenance of remission (in later assessments) will include information obtained in prior waves of data collection along with data on intensity of efforts needed to obtain outcome data in a discrete-time survival framework (i.e., in response to the 1st, 2nd, or 3rd e-requests, a subsequent telephone call appeal for response, and later telephone interviews). A range of assumptions about the distribution of the missing data will be made in this approach to investigate sensitivity of results [194]. We will record and report distributions and correlates of dropout and missing data and account for all patients in reports.

### Plans to give access to the full protocol, participant-level data, and statistical code {31c}

Only research team members from WVU and Harvard will have access to the final analytic data file. Requests for access to the full study protocol or statistical code should be directed to the PI.

## Oversight and monitoring

### Composition of the coordinating center and trial steering committee {5d}

The project Scientific Advisory Committee (SAC) will include as members PI Bossarte (Chair) and Co-PI Kessler along with collaborating researchers, clinical care providers, payers, and patient partners with lived experience. The SAC will meet by telephone and Zoom at least monthly for the first 6 months of the project and quarterly thereafter. The roles and decision-making authority of SAC members will be defined collaboratively and clearly stated, such that the first set of meetings will be devoted to establishing roles and expectations, giving each member equal time to describe what they hope to achieve or learn through the study and participation in the SAC and what they hope to offer. The SAC’s main role will be to ensure that a broad spectrum of patients and other stakeholders advise and assist the research team with refining the study questions, outcomes, and protocols.

The project Implementation Monitoring Committee (IMC) will have a similar composition as the SAC but will have the separate task of providing ongoing quality control (QC) monitoring to guarantee that recruitment of participants, intervention implementation, data collection, and report preparation-dissemination follow PCORI principals of being patient-centered. The IMC will meet by telephone and Zoom as needed and at least monthly for the first 9 months of the project and as needed thereafter to review technical assistance calls received by the project 800 number as well as any confusions or complaints to consider opportunities for improving participant experiences.

The SAC and IMC will both monitor authenticity of engagement by using and discussing PCORI’s (the funding agency) Ways of Engaging: ENgagement ACtivity Tool (WE-ENACT) Inventory during meetings, once in the initial months and at least twice annually subsequently. To uphold the PCORI Engagement Principle of *co-learning*, all researcher and clinician SAC and IMC members will seek to better understand patient populations’ needs and priorities by reviewing the commentaries of patients with lived experience created by our partners in the Depression and Bipolar Support Alliance of West Virginia.

### Composition of the data monitoring committee, its role and reporting structure {21a}

The PI along with co-investigators will have overall responsibility for monitoring the integrity of study data and participant safety. In addition, an independent monitoring committee, the Data and Safety Monitoring Board (DSMB), will be established. DSMB members will consist of (1) an expert in mental health research; (2) a clinical researcher experienced in conducting randomized clinical trials for depression; (3) three experts in assessing and treating depression; and (4) a stakeholder with immediate family members diagnosed with mood disorders. All members of the DSMB will either be established PIs, have DSMB experience, lived experience with mood disorders, and/or will be intimately familiar with the safety and ethical concerns related to human subjects in clinical research. The DSMB will review the progress of the trial and safety of participants bi-annually (i.e., two times per year), discuss any safety concerns that have arisen, and make recommendations to improve safety procedures if indicated. At each meeting, the DSMB will evaluate the progress of this project, review data quality, recruitment, and study retention and examine other factors that may affect outcome. The DSMB will review reports of any serious adverse events and/or unanticipated problems that occurred within the past study period. They will review the rates of adverse events to determine any changes in participant risk. The chair of the DSMB will report back to the AMHI investigators and will generate a brief report regarding each meeting for the study record and forwarded for review to the Institutional Review Board (IRB).

### Adverse event reporting and harms {22}

The project 800 number will be continually monitored for reports of adverse events and harms. Participants will be informed that the 800 number should be used for this purpose and solicitation of such reports will be sought as part of ongoing contacts with participants in completing SRQs. Messages reported to the study team using the 800 number are immediately delivered to designated members of the study team as an email attachment. Follow-up contact with study participants to assess safety and provide referral to crisis services if needed will occur within 24 h of message notification. Adverse events anticipated in this study include indicators of imminent risk for suicide (e.g., ideation, plans or recent attempt) or need for inpatient psychiatric hospitalization. A log of all reported adverse events, outcomes, and impact on study participation (if any) will be maintained by the study team. Adverse events will be categorized by type (behavioral, psychiatric, social, medical, etc.) and outcomes. In addition, as guided i-CBT coaches and telephone interviewers can be informed about adverse events and harms, these individuals will be instructed to notify the WVU study manager immediately of any such reports. The PI, IMC, DSMB, and IRB will all be notified immediately of each such report. Based on the judgment of the PI in consultation with the Chairs of the IMS, DSMB, and IRB, the IMC will meet as needed to discuss management strategies based on such reports.

### Frequency and plans for auditing trial conduct {23}

Study activities, including those related to consent, data collection, and participation in the interventions will be monitored on an ongoing basis by the study IMC and DSMB as well as by the WVU IRB. As noted above, the IMC will have regularly scheduled meetings by telephone and Zoom at least monthly for the first 9 months of the project and as needed thereafter to review technical assistance calls received by the project 800 number as well as any confusions or complaints to consider opportunities for improving participant experiences. The DSMB will meet twice a year and more frequently as necessary to review trial progress and participant safety.

### Plans for communicating important protocol amendments to relevant parties {25}

Changes to the study protocol will be communicated during SAC and IMC meetings and as needed to clinical partners and participants.

{26b} This study will not involve collecting biological specimens for storage.

### Dissemination plans {31a}

As noted above, the trial is being carried out in collaboration with the Depression and Bipolar Support Alliance of West Virginia (DBSAWV) [195] and the West Virginia Practice Based Research Network (WVPBRN) [196], both of which will collaborate in project dissemination activities. DBSA is the leading peer-directed organization in America focused on depression and bipolar disorder. With nearly 650 peer support groups and 250 chapters nationally, including an active chapter in West Virginia, DBSA reaches millions of people each year, offering support, referrals, and understandable information about the nature of and treatments for these disorders. DBSAWV Executive Director Diana Thompson and 4 DBSAWV members with lived experience of MDD will be members of SAC and IMC and will work closely with project researchers to disseminate results to patients and their familiar in West Virginia and, through the national DBSA, throughout the country. The PBRN has the goal of finding “real solutions for the health problems facing the people of West Virginia” and, to that end, disseminates the results of PBRN projects to healthcare providers throughout the network. In addition, the project team will prepare scientific reports of study results for publication in high-impact journals. In addition, to honor a promise we make to participants to inform them of study results, project staff will prepare and disseminate print materials summarizing study results to participants and will host a series of webinars to present and discuss results with participants.

## Discussion

The AMHI trial has the potential to be of considerable importance in addressing the problem of suboptimal treatment of MDD. We know several things that lead us to this view. First, although combined ADM-psychotherapy yields aggregate MDD remission rates about 50% higher than ADM only [19], only about 10% of MDD patients in the USA receive combined ADM-psychotherapy compared to 77% receiving ADM only [197]. Second, we know that this under-use of combined treatment is not because ADM is preferred over psychotherapy, as 75% of primary care patients with depression express a desire for psychotherapy either alone (40%) or in combination with ADM (35%) [42]. The mismatch is instead due to the low availability of psychotherapy. Given that MDD remission increases substantially when patients are not treated with their preferred type of treatment [43–45], there is good reason to believe that providing access to psychotherapy in addition to ADM will improve MDD treatment response substantially. But, third, we recognize that it is not realistic to think that this will happen in the short term due to the limited number of psychotherapy treatment providers in the country coupled with the substantially rising need and demand for treatment associated with COVID-19 [198, 199].The only realistic option is for combined treatment to be implemented using guided i-CBT added to ADM, as guided i-CBT is scalable and inexpensive. Specifically, guided i-CBT can be delivered by BA-level lay coaches, each full-time equivalent of whom can guide the treatment of well over 100 patients. Furthermore, a team of a dozen lay coaches can be supervised by a single psychotherapist, leveraging the skills of the psychotherapist to reach well over 1000 patients per week rather than the 30 a full-time psychotherapist typically treats each week in one-on-one psychotherapy. Importantly, guided i-CBT has aggregate MDD treatment effects that are comparable to those of face-to-face CBT [21, 22].

And it is clear that combined ADM-CBT is not helpful for all patients, given that the number of patients with MDD that remit with combined treatment is only proportionally 50% higher than the number that remit with ADM only. This means that at least two-thirds of patients with MDD are as likely to remit with ADM only as combined ADM-psychotherapy. Knowing which patients are which could be valuable in allocation of guided i-CBT to maxmize cost-effectiveness. In addition, if probability of remission among some patients is lower for combined treatment than for ADM only, the proportion of patients that remit with optimal allocaton would be even greater than 1.5 times the number that remit with ADM only. A similar line of thinking applies to self-guided i-CBT, which might be equally or perhaps even more effective than guided i-CBT for some patients and could be delivered at a much lower cost. All these possibilities will be examined in the AMHI trial. Results will have great potential to provide actionable information to help patients, clinicians, and payers know when to use i-CBT and at what level of intensity. It also has the potential to increase awareness of i-CBT across the USA.

## Trial status

IRB Approval of Protocol Version 1.0; 3/13/2020. Recruitment began 11/1/2020. Recruitment is tentatively scheduled to be completed 4/31/2022.

## Data Availability

Trial materials can be obtained from the first author upon request.

## Abbreviations

ADM: Antidepressant medication
AMHI: Appalachian Mind Health Initiative
CBT: Cognitive behavioral therapy
CI: Confidence interval
CV: Cross-validation
CV-TMLE: Cross-validated target minimum loss-based estimator
DBSAWV: Depression and Bipolar Support Alliance of West Virginia
DSMB: Data Safety Monitoring Board
EMR: Electronic medical record
HMS: Harvard Medical School
HRSD: Hamilton Rating Scale for Depression
HTE: Heterogeneity of treatment effects
i-CBT: Internet-based cognitive behavioral therapy
IMC: Implementation Monitoring Committee
IPW: Inverse probability weighting
ITR: Individualized treatment rule
MDD: Major Depressive Disorder
NNT: Number needed to treat
PCORI: Patient-Centered Outcomes Research Institute
PI: Principal investigator
PII: Personally identifying information
QIDS-SR: Quick Inventory of Depressive Symptomatology Self-Report
RDQ: Remission from Depression Questionnaire
SAC: Scientific Advisory Committee
SL: Super learner
SRQ: Self-report questionnaire
VHA: Veterans Health Administration
WVPBRN: West Virginia Practice Based Research Network
WVPCA: West Virginia Primary Care Association
WVU: West Virginia University
